# Initiation of Buprenorphine in the Emergency Department: A Survey of Emergency Clinicians

**DOI:** 10.5811/westjem.18029

**Published:** 2024-06-27

**Authors:** Ariana Barkley, Laura Lander, Brian Dilcher, Meghan Tuscano

**Affiliations:** * West Virginia University, Department of Emergency Medicine, Morgantown, West Virginia; † West Virginia University, Department of Behavioral Medicine and Psychiatry, Morgantown, West Virginia

## Abstract

**Introduction:**

Initiation of buprenorphine for opioid use disorder (OUD) in the emergency department (ED) is supported by the American College of Emergency Physicians and is shown to be beneficial. This practice, however, is largely underutilized.

**Methods:**

To assess emergency clinicians’ attitudes and readiness to initiate buprenorphine in the ED we conducted a cross-sectional, electronic survey of clinicians (attendings, residents, and non-physician clinicians) in a single, academic ED of a tertiary-care hospital, which serves a rural population. Our survey aimed to assess emergency clinicians’ attitudes toward and readiness to initiate buprenorphine in the ED and identify clinician-perceived facilitators and barriers. Our survey took place after the initiation of the IMPACT (Initiation of Medication, Peer Access, and Connection to Treatment) project.

**Results:**

Our results demonstrated the level of agreement that buprenorphine prescribing is within the emergency clinician’s scope of practice was inversely correlated to average years in practice (R^2^ = 0.93). X-waivered clinicians indicated feeling more prepared to administer buprenorphine in the ED R^2^ = 0.93. However, they were not more likely to report ordering buprenorphine or naloxone in the ED within the prior three months. Those who reported having a family member or close friend with substance use disorder (SUD) were not more likely to agree buprenorphine initiation is within the clinician’s scope of practice (*P* = 0.91), nor were they more likely to obtain an X-waiver (*P* = 0.58) or report ordering buprenorphine or naloxone for patients in the ED within the prior three months (*P* = 0.65, *P* = 0.77). Clinicians identified availability of pharmacists, inpatient/outpatient referral resources, and support staff (peer recovery support specialists and care managers) as primary facilitators to buprenorphine initiation. Inability to ensure follow-up, lack of knowledge of available resources, and insufficient education/preparedness were primary barriers to ED buprenorphine initiation. Eighty-three percent of clinicians indicated they would be interested in additional education regarding OUD treatment.

**Conclusion:**

Our data suggests that newer generations of emergency clinicians may have less hesitancy initiating buprenorphine in the ED. In time, this could mean increased access to treatment for patients with OUD. Understanding clinician-perceived facilitators and barriers to buprenorphine initiation allows for better resource allocation. Clinicians would likely further benefit from additional education regarding medications for opioid use disorder (MOUD), available resources, and follow-up statistics.

Population Health Research Capsule What do we already know about this issue?

*Initiation of buprenorphine in the Emergency Department (ED) for opioid use disorder (OUD) has been shown to be beneficial, but is largely underutilized. *

What was the research question?

*What are clinicians’ attitudes toward initiating buprenorphine in the ED, and what are the barriers to prescribing? *

What was the major finding of the study?

*Clinician likelihood of initiating treatment in the ED was inversely correlated to years in practice. The primary barrier to initiating buprenorphine was inability to ensure follow-up. *

How does this improve population health?

*Eliminating barriers and improving clinician readiness to initiate buprenorphine in the ED could increase access to care for patients with OUD.*

## INTRODUCTION

More than 564,000 individuals died of opioid overdose in the US from 1999–2020,^1^ according to the US Centers for Disease Control and Prevention; more recent, provisional data suggests that annual overdose rates continued to rise in 2021.^2^ As would be expected, with increased rates of overdose, emergency department (ED) visits for opioid overdose also increased in 2020.^3^ Patients with opioid use disorder (OUD) are frequently seen in the ED with both overdose and other less emergent conditions. Patients seen in the ED after a non-fatal opioid overdose have >5% one-year mortality rat.^4^ The ED is a low-barrier access point to the healthcare system, and ED visits represent a valuable opportunity to engage patients with OUD in potentially lifesaving treatment.

Buprenorphine, a US Food and Drug Administration (FDA)-approved medication for OUD (MOUD), has been shown to be effective in decreasing overall opioid use, reducing risk of opioid overdose, and reducing both opioid-associated and all-cause mortality.^5^ Buprenorphine has been available to emergency clinicians for the treatment of opioid withdrawal since 2002, and research has shown the benefits of buprenorphine initiation in the ED.^6^ Specifically, in comparison to referral to treatment or brief ED intervention, initiation of buprenorphine in the ED results in increased rates of engagement in addiction treatment at 30 days and decreased illicit opioid use.^7^ The American College of Emergency Physicians (ACEP) recommends the initiation of buprenorphine in appropriate patients. Additionally, the ACEP consensus states: “Detecting and offering evidenced-based treatments for patients with opioid use disorder is aligned with the goals of emergency medicine to intervene on high-mortality disease processes.”^8^

Unfortunately, MOUDs including buprenorphine are largely underutilized, and the majority of people with OUD do not received treatment with MOUDs.^9^ Substance use disorders (SUD) are one of the most highly stigmatized medical conditions in the world among clinicians and the general public.^10^^,^^11^ A study looking at emergency physicians’ attitudes toward patients with SUD found that emergency physicians had a lower regard for patients with SUD than other medical conditions with behavioral components.^12^ The MOUDs, including buprenorphine, are also stigmatized, which impacts treatment access and prescribing practices for these medications.^13^ Previous findings identify the most significant barriers to prescribing buprenorphine in the ED include logistical or systemic factors as well as perceived patient factors (ie, social barriers and lack of interest in treatment).^14^ Clinician lack of knowledge, as well as their attitudes and biases, can impact willingness to prescribe medications such as buprenorphine for patients with OUD, despite MOUD being a well studied and effective treatment.^6^^,^^15^ Not only are patients on MOUD stigmatized but the prescribers who provide them with medications are also stigmatized.^16^

To promote engagement in and referral to treatment for OUD, our academic ED initiated the IMPACT project (Initiation of Medication, Peer Access, Connection to Treatment) in 2020. Key elements of the IMPACT project included electronic health record (EHR) prompts and order sets, peer recovery support specialists in the ED, and availability of inpatient and outpatient referral, all of which are barriers identified in previous studies.^15^^,^^17^^–^^18^ Additionally, when the IMPACT project was introduced to the ED, clinicians were offered a financial incentive to obtain a US Drug Enforcement Administration X-waiver. The primary goal of our study was to assess emergency clinicians’ attitudes toward and readiness to initiate buprenorphine in the ED, as well as identify perceived facilitators and barriers to initiating buprenorphine treatment in an academic ED, after implementation of the IMPACT project and its associated resources.

## METHODS

This study was part of a State Opioid Response Implementation project called IMPACT. The primary objective of the project was to integrate peer recovery support specialists (PRSS) in the ED, to increase buprenorphine prescribing for patients with OUD, and to increase engagement and referrals to treatment for all patients with SUD. We extracted data from the EHR regarding patient demographics, PRSS interaction with patients, and prescribing practices over a two-year period from March 2020–March 2022. A mixed-methods model was used to evaluate the data. This project was approved by the institutional review board.

We conducted a cross-sectional electronic-based survey regarding buprenorphine prescribing in the ED with all potential ED prescribers including attending physicians, resident physicians, physician assistants, and nurse practitioners. We developed the survey, adapting from previously published research.^15^^,^^17^^–^^18^ Prior surveys had been conducted in large urban areas but had not been deployed in a more rural setting. Our survey was designed to identify prescribers’ attitudes toward and readiness to initiate buprenorphine in the ED and identify perceived facilitators and barriers to initiating buprenorphine treatment in an academic ED of a large, tertiary-care hospital, which serves a rural population. Clinicians were made aware of the study through an initial email, two email reminders, a one-time announcement at our weekly didactic conference, and flyers posted throughout the ED. Participants were incentivized, as the first 100 participants received a $10 gift card, and all participants were entered for a chance to win a $100 gift card.

The survey completed by emergency clinicians included 10 questions focusing on years in practice, X-waiver status, prescribing practices in the ED in the prior three months, comfort with treatment of OUD and prescribing buprenorphine in the ED, and personal experience with SUD. Two additional Likert-scale questions assessed for barriers and facilitators to prescribing buprenorphine. (See Appendix A for full survey). The survey was published March 23, 2022, and closed May 15, 2022. Survey responses were recorded via Qualtrics (Qualtrics, Provo, UT), and the data was exported to a secure Excel file (Microsoft Corp, Redmond, WA) for analysis. We then organized and analyzed the data using SAS 9.4 (SAS Institute Inc, Cary, NC) with chi-squared or Fisher exact tests. We de-identified and extracted additional operational patient data on the IMPACT program on a rolling basis from the EHR.

## RESULTS

A total of 95 surveys were distributed to all emergency clinicians (attending physicians, residents, physician assistants, and nurse practitioners) There were a total of 43 respondents and a response rate of 45% (16/50 attendings, 21/30 residents, 6/15 physician assistants and nurse practitioners). Three surveys were partially completed. We included two that had >50% of the questions answered and excluded one survey with only two questions completed as the latter respondent’s intent to complete was interpreted as questionable. Of those who responded, their years in practice ranged from 1-50 with an average of 7.3 years. Of the 43 respondents, 31 indicated they were familiar with the IMPACT project and 12 said they were not. All the respondents who indicated they were not familiar with the IMPACT project were ED residents. (See Tabl.) Notably, 83% of all respondents indicated they would be interested in additional education related to medication and resources for OUD treatment.

**Table.** Data summary of emergency clinicians who participated in a survey regarding ED-initiated buprenorphine.

	Count	Percentage
Participants (total)	42	
Attending physicians	16	38.1%
Non-physician clinicians	6	14.3%
Residents	20	47.6%
Years in practice		
Minimum	1	
Maximum	50	
Average	7.31	
Median	4	
Familiar with IMPACT		
Yes	31	73.8%
No	11	26.2%
X-waivered		
Yes	16	38.1%
No	26	61.9%
Family/friend with substance use disorder		
Yes	18	42.9%
No	24	57.1%

*IMPACT*, initiation of medication, peer access, and connection to treatment.

**Figure 1.**
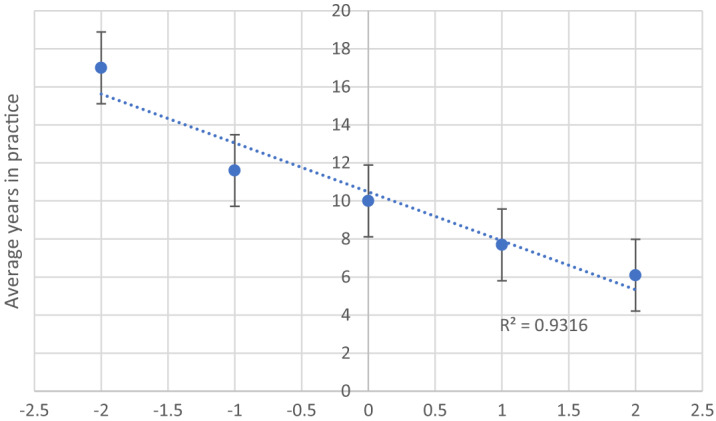
Agreement that buprenorphine is within the emergency clinician’s scope of practice as assessed on a 5-point Likert scale in comparison to average years in practice.

A five-point Likert scale was used to assess respondents’ level of agreement that prescribing buprenorphine was within their scope of practice. While 78.6% of respondents agreed that prescribing buprenorphine was within their scope, the level of agreement was found to be inversely correlated with average years in practice (R^2^ = 0.93162) (Figure 1). Regarding X-waiver status, 16 individuals identified as having their X-waiver and 26 indicated they were not X-waivered. When asked why they were not waivered, four individuals indicated they were “not interested,” three said cost was a barrier, seven said time was a barrier, and 12 responded “other.” In the “other” category, two responded they were unsure how to obtain the waiver; two questioned whether it was needed; one said “in the process”; three said “just haven’t done it”; one indicated they had completed the training but were not yet licensed; and one said “I know the data shows it works, but I still feel like a drug dealer.” We found that those who had an X-waiver, in comparison to those who did not, were more likely to feel prepared to administer buprenorphine in the ED (*P* = 0.02).

To enable us to describe prescribing practices, prescribers were also asked whether they had ordered naloxone for patients in the ED in the prior three months; 29 said “yes” and 13 said “no.” When asked whether they had ordered buprenorphine for patients in the ED in the prior three months, 18 said “yes” and 24 said “no.” We also observed that those who had an X-waiver were not more likely to have reported ordering buprenorphine or naloxone for patients in the ED within the prior three months (*P* = 0.17), (*P* = 0.51).

**Figure 2.**
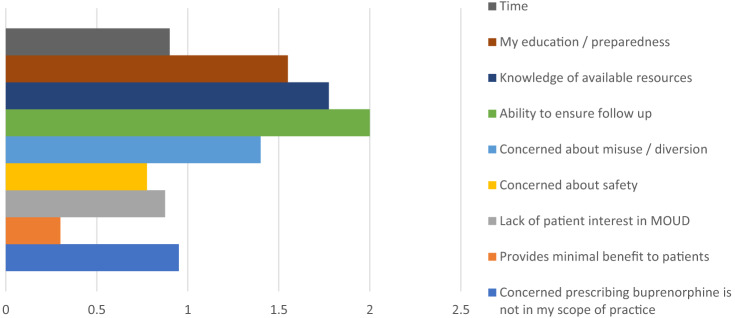
Clinician-perceived barriers to initiating buprenorphine in the emergency department. Identified barriers were graded with a 3-point Likert scale: somewhat a barrier, moderate barrier, significant barrier. *MOUD*, medication for opioid use disorder.

**Figure 3.**
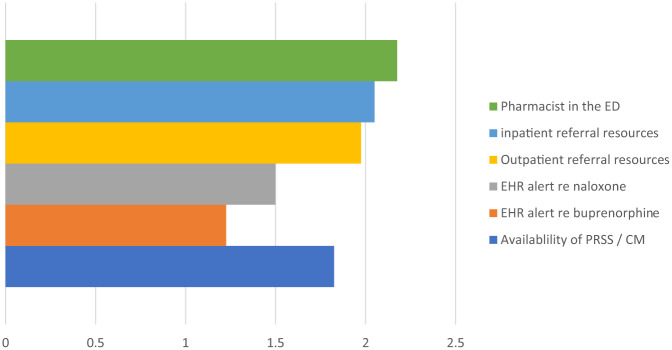
Clinician-perceived facilitators to initiating buprenorphine in the emergency department. Identified facilitators were graded with a 3-point Likert scale: somewhat a facilitator, moderate facilitator, significant facilitator. *ED*, emergency department; *EHR*, electronic health record; *PRSS*, peer recovery support specialist; *CM*, case manager.

Sixty-seven percent of clinicians agreed that they felt prepared to administer buprenorphine in the ED, 53.7% agreed that they felt prepared to prescribe buprenorphine as a bridge to outpatient treatment, and 47.6% agreed that they felt prepared to prescribe buprenorphine for home induction. Sixty-two percent of all respondents agreed that they had all the resources needed to initiate buprenorphine in the ED. Barriers and facilitators to initiating buprenorphine in the ED are identified in Figure 2 and Figure 3, respectively.

To assess possible personal barriers and facilitators of buprenorphine prescribing the following was asked: “Have you had, or do you currently have a family member or close friend with SUD?” Responses indicated 43% said “yes” and 57% said “no.” Those who reported having a family member or close friend with SUD were not more likely to 1) agree that buprenorphine initiation is within the emergency clinician’s scope of practice (*P* = 0.91); 2) obtain an X-waiver (*P* = 0.58); or 3) report ordering buprenorphine or naloxone for patients in the ED within the prior three months (*P* = 0.65), (*P* = 0.77).

### IMPACT Project Qualitative Results

Over the two-year period, 1,205 patients were seen in the ED by PRSSs, 13% of whom were diagnosed with OUD or opioid withdrawal. A total of 377 were referred for buprenorphine treatment by the PRSSs within the ED; 168 of those patients received buprenorphine treatment, and 42 were given a take-home prescription. At the start of the study there were three X-waivered physicians; during the course of the project, 12 additional clinicians obtained their X-waiver, for a total of 15.

## DISCUSSION

Our survey aimed to evaluate emergency clinicians’ attitudes toward and preparedness to initiate buprenorphine in the ED as well as identify perceived facilitators and barriers to initiating buprenorphine treatment after the implementation of the IMPACT project and its associated resources. Our results showed that 78.6% of clinicians agreed that prescribing buprenorphine in the ED was within their scope of practice. As shown in Figure 1, the level of agreement that buprenorphine is within the emergency clinician’s scope of practice was inversely correlated to years in practice. Another study found that clinicians with fewer years in practice were more likely to believe that OUD is like other chronic diseases and were more likely to approve of ED-initiated buprenorphine.^18^ Other studies have identified emergency medicine residents as enthusiastic and eager to incorporate care for OUD into their practice.^17^^,^^19^ We believe these results are encouraging and demonstrate that newer generations of clinicians may have less hesitancy toward initiating MOUD treatment in the ED setting. This change will, in time, likely increase access to care for those with OUD.

Sixty-seven percent of all clinicians agreed that they felt prepared to administer buprenorphine in the ED. We suspect clinicians’ level of preparedness could be improved with continuing education lectures and feedback. Notably, the majority of respondents reported they would be interested in additional education related to medication and resources for OUD treatment.

We found that those with an X-waiver, in comparison to those who did not have an X-waiver, were more likely to feel prepared to administer buprenorphine in the ED. Other studies have found that X-waivered clinicians reported higher levels of readiness or preparedness to initiate buprenorphine in the ED in comparison to those who were not X-waivered.^14^^,^^17^ Previously, an eight-hour training course was required to obtain an X-waiver; this training requirement, and the hassle of obtaining a waiver, was previously identified as a barrier to initiating buprenorphine in the ED.^14^^,^^17^^–^^18^^,^^20^ However, finding that X-waivered clinicians felt more prepared to administer buprenorphine in the ED may reflect the value that was associated with the previously required education course. Notably, we also found that those who had an X-waiver were not more likely to have reported ordering buprenorphine or naloxone for patients in the ED within the prior three months. This finding potentially supports the idea that simply increasing the number of X-waivered clinicians does not significantly improve access to care if X-waivered clinicians are not actively prescribing MOUDs.^21^^,^^22^ Notably, our data was collected prior to the recent elimination of the national X-waiver requirement.

When we asked whether having had a friend or family member with SUD would affect clinicians’ attitudes toward buprenorphine in the ED, we found that 42.8% of clinicians reported having had a family member or close friend with SUD. This personal relationship, however, did not make clinicians statistically more likely to 1) agree that prescribing buprenorphine was within the emergency clinician’s scope of practice; 2) obtain an X-waiver; or 3) report ordering buprenorphine or naloxone for patients in the ED within the prior three months. To our knowledge, a prescriber’s personal relationships to individuals with SUD has not been evaluated in prior studies.

Sixty-two percent of clinicians indicated they have the resources they need to initiate buprenorphine in the ED. With the IMPACT project, as described above, clinicians have resources such as peer recovery support specialists in the ED, EHR prompts, and close outpatient follow-up available. Additionally, our academic ED is staffed with pharmacists and case managers/social workers 24/7. Given the number of resources available, we would have expected that more clinicians would have felt they have the resources necessary to initiate buprenorphine in the ED. We suspect it is possible that many clinicians felt they did not have the resources necessary because they were simply unaware of the available resources. Notably, less than 75% of respondents were familiar with the IMPACT project. All of those who were unfamiliar with the IMPACT project were residents; this highlights an opportunity for additional education.

A number of studies have been conducted looking at facilitators and barriers to buprenorphine initiation in the ED.^14^^,^^17^^–^^18^ Previously identified barriers to initiating buprenorphine in the ED include the following: lack of training/experience; concerns regarding misuse/diversion/harm; patient interest; time/competing priorities in the ED; concerns regarding follow-up; concerns regarding increased ED volume; and feeling as if prescribing buprenorphine was not within their scope of practice.^14^^,^^17^^–^^18^

Notably, with the implementation of the IMPACT project and its associated resources, several systemic/logistical barriers have been eliminated as PRSSs are available in the ED, outpatient follow-up can be ensured, and the EHR is equipped with prompts and order sets regarding both buprenorphine and outpatient referrals.

Our clinicians identified inability to ensure follow-up, limited knowledge of available resources, and lack of education/preparedness as the top three barriers to initiating buprenorphine in the ED. Although the COAT (comprehensive opioid addiction treatment) clinic has a standing appointment for ED referrals, and PRSSs work to facilitate these appointments, and even accompany patients to these appointments, concern regarding follow-up was still the primary barrier identified by clinicians. A recent study validated these concerns as it found that less than 30% of patients who fill buprenorphine prescriptions from the ED fill subsequent buprenorphine prescriptions.^23^ Currently we do not have data regarding ED follow-up rates or rates of subsequent buprenorphine refills; however, this is an area of interest for future investigation to better evaluate the effectiveness of our IMPACT program.

Previously identified facilitators to buprenorphine initiation in the ED include ability to ensure follow-up; support staff – PRSSs/social work/care managers; department protocols; EHR order sets; pharmacist consultation; and feedback on patient experiences.^14^^,^^17^^–^^18^ Our clinicians identified availability of pharmacists and of both inpatient and outpatient resources, and the presence of PRSSs and care managers as primary facilitators to buprenorphine initiation in the ED. The fact that clinicians identified pharmacist availability as a significant facilitator likely highlights underlying clinician discomfort with the pharmacology of buprenorphine and again highlights an opportunity for ongoing education and experience. Notably, time was not a primary barrier identified by our clinicians, and this may be due to the presence of additional support staff in the ED.

## LIMITATIONS

Our study has several limitations. Overall we had a small sample size, and our respondents all work at the same academic center. Additionally, nearly half of respondents were residents with fewer than three years in clinical practice. Our data was collected prior to the elimination of the X-waiver requirement. It is possible that this new legislation has since influenced prescribers’ attitudes toward buprenorphine as well as prescribing practices. Results related to facilitators and barriers may not be generalizable to community-based, non-academic EDs that do not have similar resources. Additionally, our results may not be generalizable to academic EDs in urban areas.

## CONCLUSION

The results of our survey identified the following: 1) agreement that buprenorphine is within the emergency clinician’s scope of practice was inversely correlated to years in practice; 2) >80% of clinicians were interested in additional education regarding MOUDs and resources for OUD treatment; 3) those with an X-waiver were more likely to report feeling more prepared to administer buprenorphine in the ED in comparison to those who were not X-waivered; and 4) clinicians who reported having had a family member or close friend with SUD were not more likely to agree that buprenorphine initiation is within the emergency clinician’s scope of practice, nor were they more likely to obtain an X-waiver or report ordering buprenorphine or naloxone for patients in the ED within the prior three months. We also identified clinician-perceived barriers and facilitators to initiating buprenorphine in the ED. Our clinicians identified inability to ensure follow-up as a primary barrier to initiating buprenorphine in the ED.

More research is needed on retention in treatment following ED referral to identify what factors are associated with successful transitions of care from ED-initiated MOUD to community-based treatment. Education/preparedness was also identified as a significant barrier. We plan to address this with additional didactics and program updates. Time was less of a barrier, likely secondary to the availability of pharmacists, support staff, and inpatient and outpatient resources, which were identified as facilitators. A better understanding of facilitators and barriers allows for better resource allocation.

## Supplementary Information




