# Supplementary material for: Initiation of Buprenorphine in the Emergency Department: A Survey of Emergency Clinicians
Source: West J Emerg Med. 2024 Jun 27;25(4):470–6. doi: 10.5811/westjem.18029 (PMC11254164; doi:10.5811/westjem.18029)
Supplement: Supplementary file 1 [file wjem-25-470-s001.docx]

To participate in the study please select "I agree" below to provide your electronic consent as well as acknowledgement that you have read the cover letter and understand the contents.

- I agree
- I do not agree

End of Block: Cover Letter & Consent

Start of Block: IMPACT Peer Acceptability

What is your role in the Emergency Department?

- APP
- Attending Physician
- Clinical Assistant
- ED Tech
- Mental Health Specialist
- Nurse
- Pharmacist
- Resident Physician
- Social Work/Care Management
- Unit Clerk
- Other __________________________________________________

Are you familiar with the IMPACT project?

- No
- Yes

Were you working/employed in the Ruby ED when the IMPACT project was initiated on 3/10/2020?

- No
- Yes

| 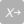 |
| --- |

Please rate your interactions with the Peer Recovery Support Specialists (PRSS) on the IMPACT Project using the following scale:

|  | Extremely Negative | Somewhat negative | Neither positive nor negative | Somewhat positive | Extremely positive | N/A  (I have not interacted with a PRSS) |
| --- | --- | --- | --- | --- | --- | --- |
| Rate your interactions with the Peer Recovery Support Specialist (PRSS) on the IMPACT project. |  |  |  |  |  |  |

In what ways, if any, do you think having PRSS in the ED has helped patient care/outcomes or ED flow? (select all that apply)

- Support for patients
- Reduced my workload
- Reduced patient length of stay
- Helped me better understand substance use disorders
- Helped reduce stigma against people with substance use disorders
- Help give providers hope that people with substance use disorders get better
- Other __________________________________________________
- None of the above

In what ways, if any, do you think having PRSS in the ED has been unhelpful to patient care/outcomes or ED flow? (select all that apply)

- I cannot always find the PRSS
- Patients become difficult to manage after they talk to the PRSS
- PRSS interrupt my workflow
- PRSS are unprofessional
- Other __________________________________________________
- None of the above

What do you consider strengths of the IMPACT project? (select all that apply)

- Has improved referrals to residential/detox settings
- Improved patient access to Buprenorphine in the ED to treat opioid withdraw
- More streamlined connection to outpatient treatment (COAT clinic)
- PRSS have helped patients determine what treatment is most appropriate for their current situation
- PRSS interventions with patients in the ED in real time
- Other __________________________________________________

How do you think the IMPACT project could be improved?

________________________________________________________________

________________________________________________________________

________________________________________________________________

________________________________________________________________

________________________________________________________________

End of Block: IMPACT Peer Acceptability

Start of Block: Prescriber Questions

| 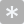 |
| --- |

How many years do you have in practice? (In years, round up to the nearest whole number - include residency years)

________________________________________________________________

Have you obtained your DEA X-Waiver to prescribe Buprenorphine?

- No
- Yes

When was your X-waiver issued? (Month and Year)

________________________________________________________________

If no, why?

- Not interested
- Takes too much time
- Costs too much
- Other __________________________________________________

Have you had or do you currently have a close friend or family member with Substance Use Disorder?

- No
- Yes

If so, are they in recovery, still actively using substances, or deceased due to their substance use?

- In recovery
- Still actively using
- Deceased due to their substance use
- Unknown

Have you ordered naloxone for patients in the ED in the past 3 months?

- No
- Yes

Have you ordered buprenorphine for patients in the ED in the past 3 months?

- No
- Yes

| Page Break |  |
| --- | --- |

For each of the following statements please respond with your level of agreement or disagreement:

|  | Strongly disagree | Somewhat disagree | Neither agree nor disagree | Somewhat agree | Strongly agree |
| --- | --- | --- | --- | --- | --- |
| Prescribing buprenorphine in the ED is within my scope of practice. |  |  |  |  |  |
| I feel that I have all of the resources I need to initiate buprenorphine in the ED - support staff (PRSSs, CM), pharmacy, treatment referral resources and follow-up pathway |  |  |  |  |  |
| I feel prepared to discuss and explain medications to treat opioid use disorder (OUD) with patients. |  |  |  |  |  |
| I feel prepared to administer buprenorphine in the ED. |  |  |  |  |  |
| I feel prepared to give patients in the ED for OUD a take home prescription of buprenorphine to bridge them to their clinic appointment. |  |  |  |  |  |
| I feel prepared to give patients in the ED for OUD a take home prescription of buprenorphine for home induction. |  |  |  |  |  |
| I would be interested in additional education related to medication and resources for OUD treatment. |  |  |  |  |  |

| Page Break |  |
| --- | --- |

How frequently do you:

|  | Multiple times per week | Weekly | Monthly | Yearly | Never |
| --- | --- | --- | --- | --- | --- |
| See a patient who asks for treatment for OUD |  |  |  |  |  |
| Refer a patient to inpatient, residential or detox treatment for OUD (via CM or psychiatry) |  |  |  |  |  |
| Refer to outpatient OUD treatment (COAT or other outpatient treatment) |  |  |  |  |  |
| Prescribe naloxone |  |  |  |  |  |
| provide a single dose of buprenorphine in the ED |  |  |  |  |  |
| Prescribe buprenorphine for home induction |  |  |  |  |  |

| Page Break |  |
| --- | --- |

Identify which of the following factors you feel are barriers to prescribing buprenorphine.

|  | Not a barrier | Somewhat a barrier | Moderate barrier | Significant barrier |
| --- | --- | --- | --- | --- |
| Concerned that prescribing/providing buprenorphine is not in my scope of practice |  |  |  |  |
| Provides minimal benefit to patients |  |  |  |  |
| Lack of patient interest in MAT |  |  |  |  |
| Concerned about safety of MAT |  |  |  |  |
| Concerned about misuse/diversion |  |  |  |  |
| Ability to ensure follow-up |  |  |  |  |
| Knowledge of available referral resources |  |  |  |  |
| My education/preparedness |  |  |  |  |
| Time |  |  |  |  |

Identify which factors you feel are facilitators to prescribing buprenorphine.

|  | Not a facilitator | Somewhat a facilitator | Moderate facilitator | Significant facilitator |
| --- | --- | --- | --- | --- |
| Availability of PRSSs/CM |  |  |  |  |
| Epic alert for elevated COWS prompting me to order buprenorphine |  |  |  |  |
| Epic alert reminding me to order nalaxone |  |  |  |  |
| Access to outpatient referral resources such as COAT clinic |  |  |  |  |
| Access to residential or inpatient referral resources |  |  |  |  |
| Availability of Pharmacist in the ED |  |  |  |  |

End of Block: Prescriber Questions

Start of Block: Block 3

Thank you for completing the survey! 


In order for us to award you a gift card for participation we need a couple demographic items from you. Upon clicking the next page button you will be re-directed to a separate survey. The first 100 respondents will receive a $10 prepaid Master Card gift card and all respondents will be entered to win a $100 prepaid Master Card gift card. 


Your responses to the following survey are not linked to your responses from this survey in any way.

End of Block: Block 3
